# Clinical Significance of Serum Omega-3 Fatty Acids on Endothelial Function in Patients with Coronary Artery Disease Under Statin Therapy

**DOI:** 10.3390/jcdd12020060

**Published:** 2025-02-05

**Authors:** Kei Yunoki, Hiroaki Matsumi, Toru Miyoshi, Motoki Kubo, Yoshiki Hata, Shinsuke Yuasa

**Affiliations:** 1 Department of Cardiovascular Medicine, Okayama University Graduate School of Medicine, Okayama 700-8558, Japan; 2 Department of Cardiology, Tsuyama Chuo Hospital, Tsuyama 708-0841, Japan; 3 Minamino Cardiovascular Hospital, Hachioji 192-0918, Japan

**Keywords:** coronary artery disease, endothelial function, eicosapentaenoic acid, residual risk factor

## Abstract

Vascular endothelial function plays an important role in the pathogenesis of atherosclerosis. The reduction in low-density lipoprotein cholesterol (LDL-C) is a key therapy for preventing coronary artery disease (CAD), but the role of omega-3 fatty acids as residual risk factors of CAD remains controversial. We studied the correlation between serum omega-3 fatty acid levels and endothelial function in patients with CAD receiving statin therapy and examined the effect of eicosapentaenoic acid (EPA) therapy on endothelial function. Methods: A total of 150 consecutive patients with CAD receiving statin therapy (LDL-C levels < 100 mg/dL) were enrolled. Serum omega-3 fatty acid levels were measured, and endothelial function was assessed by flow-mediated dilation (FMD) of the brachial artery. Subsequently, 65 patients with impaired FMD (<6%) and low EPA/arachidonic acid (AA) (<0.3) were administered EPA, and FMD was reassessed after 3 months. Results: A multivariate linear regression analysis demonstrated that serum docosahexaenoic acid (DHA) and EPA plus DHA levels were independent determinants of %FMD (β = 0.214 and 0.163, *p* < 0.05, respectively). The EPA therapy significantly improved %FMD (from 3.7 ± 1.0% to 4.1 ± 1.0%, *p* < 0.05) in patients with low EPA/AA, and especially in patients with low EPA/AA and high triglyceride levels (from 3.4 ± 1.0% to 4.0 ± 1.1%, *p* < 0.01). Conclusions: Serum omega-3 fatty acid levels were associated with endothelial dysfunction in patients with CAD receiving statin therapy. EPA therapy improves endothelial function in patients with low EPA/AA, especially those with low EPA/AA and high triglycerides.

## 1. Introduction

Statin therapy is the cornerstone of the treatment of dyslipidemia and prevention of coronary artery disease (CAD), which remains a leading cause of morbidity and mortality worldwide [1]. Statins reduce low-density lipoprotein cholesterol (LDL-C) and numerous large-scale clinical trials have consistently demonstrated that statin therapy reduces the risk of major cardiovascular events, including myocardial infarction, stroke, and cardiovascular death, in patients with established CAD or those at high risk [2,3,4]. By stabilizing atherosclerotic plaques, improving endothelial function, and reducing inflammation, statins contribute to both the primary and secondary prevention of cardiovascular disease [5]. However, despite the reduction in LDL-C, a substantial number of CAD events have not been prevented [1], emphasizing the presence of residual risks that are not addressed by LDL-C reduction alone. This unmet clinical need has driven the exploration of adjunctive therapeutic strategies to further mitigate cardiovascular risk.

Emerging evidence has suggested that omega-3 polyunsaturated fatty acids (PUFAs), particularly from fish oil, could play a crucial role in mitigating these residual risks [6]. Fish oil supplementation is inversely correlated with cardiovascular morbidity and mortality and is gaining significant attention as a therapeutic approach for residual risks [7]. Omega-3 PUFAs including eicosapentaenoic acid (EPA) and docosahexaenoic acid (DHA) lower triglyceride levels and significantly decrease postprandial triglyceride elevation and postprandial endothelial dysfunction in healthy individuals [8]. Various clinical trials have explored the efficacy of omega-3 PUFAs in different dosages and combinations, with a particular focus on EPA and DHA [6]. Despite this evidence, recent large-scale omega-3 PUFA trials (REDUCE-IT [Reduction of Cardiovascular Events with Icosapent Ethyl–Intervention Trial], STRENGTH [Long-Term Outcomes Study to Assess Statin Residual Risk Reduction With Epanova in High Cardiovascular Risk Patients With Hypertriglyceridemia], and RESPECT-EPA [Randomized Trial for Evaluation in Secondary Prevention Efficacy of Combination Therapy–Statin and Eicosapentaenoic Acid]) have sparked controversy concerning the relevance of EPA intervention [9,10,11].

Vascular endothelial function plays an important role in the pathogenesis of atherosclerosis and plaque instability [12,13]. Brachial artery flow-mediated dilation (FMD), a noninvasive ultrasound-based assessment method, has emerged as a reliable indicator of nitric oxide-dependent endothelial function [12]. FMD involves measuring the vasodilatory response of the brachial artery to increased blood flow, which reflects the ability of the endothelium to produce and release NO [14]. FMD is a prognostic surrogate marker for predicting future cardiovascular events, and the persistent impairment of FMD despite optimal medical therapy has demonstrated a negative predictive value for outcomes in patients with CAD [15], even in the era of drug-eluting stents [16]. This predictive value remains significant even in the era of advanced interventions, emphasizing the potential of FMD as a surrogate marker for cardiovascular risk stratification and therapeutic monitoring.

Accordingly, this study aimed to investigate the associations between serum omega-3 fatty acid levels and endothelial function in patients with CAD receiving statin therapy and LDL-C levels (<100 mg/dL) in patients with CAD. We also investigated the effect of EPA therapy with a statin on endothelial function in patients with low EPA/arachidonic acid (AA).

## 2. Materials and Methods

### 2.1. Study Design and Participants

This prospective, single-center, single-arm study was approved by the ethics committee of Okayama University Graduate School of Medicine, Dentistry, and Pharmaceutical Sciences (1608-005, 2016-08-01). Written informed consent was obtained from all the participants before the procedure. The study was conducted according to the principles outlined in the Declaration of Helsinki.

**Figure 1:**
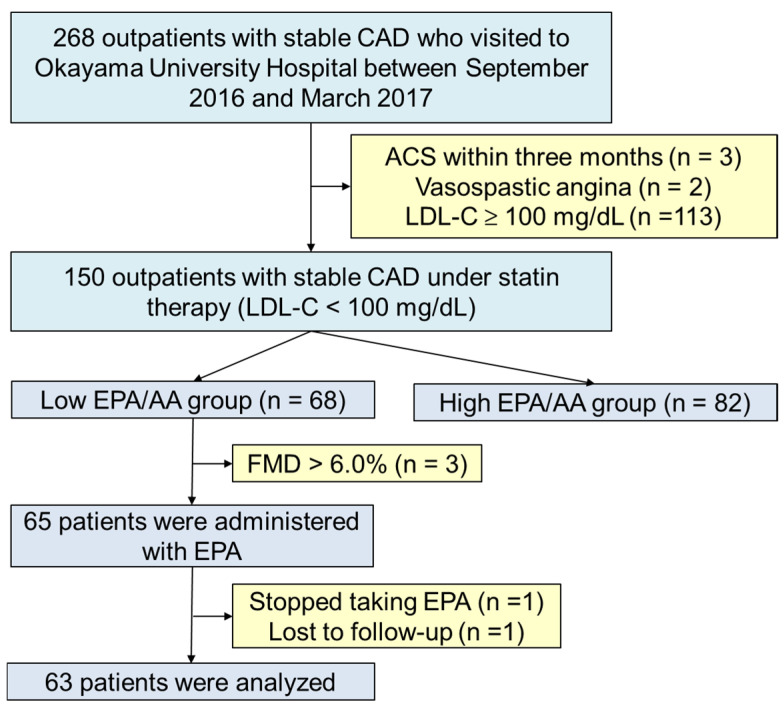
Flow diagram of this study. A total of 150 patients were included in the analysis to assess the correlation between EPA/AA with biochemical variables. Additionally, 65 patients were enrolled in the randomized study, and 63 patients were finally analyzed. CAD, coronary artery disease; ACS, ascute coronary syndrome; EPA, eicosapentaenoic acid; AA, arachidonic acid.

Of the 268 outpatients with stable CAD who visited Okayama University Hospital between September 2016 and March 2017, 150 consecutive patients had undergone percutaneous coronary intervention or coronary bypass surgery, had been receiving statin therapy for hypercholesterolemia for more than 3 months, and had achieved the target LDL-C levels (<100 mg/dL) for secondary prevention according to the 2013 Japan Atherosclerotic Society (JAS) Guideline [17] Figure 1. Patients were excluded from the study if they had acute coronary syndrome, stroke, heart failure, familial hypercholesterolemia, or had undergone major surgery within 3 months before enrollment. Additional exclusion criteria included vasospastic angina, concomitant inflammatory diseases, ongoing EPA therapy, or malignant tumors.

Among the enrolled patients, those with impaired FMD (<6%) and low EPA/AA ratio (<0.3) or high triglyceride (> 150 mg/dL) were administered EPA at 1800 mg/day orally. Lipid profiles and endothelial function were assessed at baseline and after 3 months. CAD, coronary artery disease; LDL-C, low-density lipoprotein cholesterol; FMD, flomediated dilation; EPA, eicosapentaenoic acid; AA, arachidonic acid.

### 2.2. Definition of Risk Factors

Hypertension was diagnosed as systolic blood pressure ≥ 140 mmHg or diastolic blood pressure ≥ 90 mmHg or through concurrent treatment with anti-hypertensive medication. Diabetes was defined as a fasting blood glucose level ≥ 126 mg/dL, HbA1c ≥ 6.5%, or requiring anti-diabetes medication.

### 2.3. Biochemical Analysis

Venous blood samples were collected from all the patients during their outpatient clinic visits after overnight fasting for at least 8–12 h. The levels of serum PUFAs, including EPA, DHA, AA, and dihomo-gamma-linolenic acid, were measured using a gas–liquid chromatograph (GC-2010, Shimazu, Kyoto, Japan) equipped with a capillary column (TC-70, GL Sciences, Tokyo, Japan) at SRL Co. Ltd., Tokyo, Japan [8]. The other parameters were serum triglycerides, LDL-C, high-density lipoprotein (HDL-C), uric acid (UA), fasting plasma glucose, hemoglobinA1c, and high-sensitivity C-reactive protein. The lipid profiles and other markers were analyzed at SRL Co. Ltd., Tokyo, Japan.

### 2.4. Assessment of Endothelial Vasomotor Function

**Figure 2:**
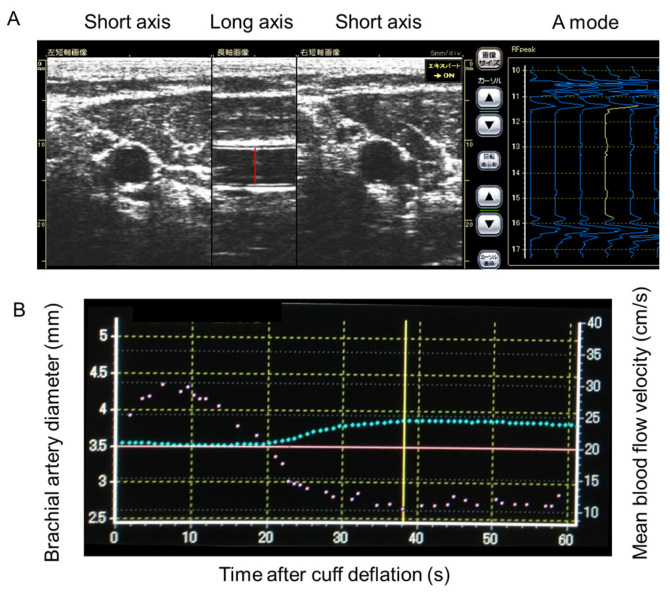
Assessment of flow mediated dilation (FMD) with UNEXEF18G. (**A**) The H-shaped probe of the UNEXEF18G shows 2 short-axis and 1 long-axis images of the brachial artery (left) and both the near and far walls of the brachial artery as A-mode ultrasound signals (rt). The red line indicates diameter of the brachial artery. (**B**) Representative images of the change in brachial artery diameter after cuff deflation to measure FMD. The green dot line and red dot line indicate the changes in brachial artery diameter and blood flow velocity, respectively. FMD is measured at the point indicated by the yellow line.

Endothelium-dependent dilations were assessed as parameters of vasodilation according to the guidelines for the ultrasound assessment of FMD of the brachial artery [14]. The assessments were performed with equipment specialized for FMD measurements (UNEXEF18G, UNEX Co, Nagoya, Japan). Briefly, the diameter of the brachial artery was measured from B-mode ultrasound images using an attached 10 MHz linear array transducer. The brachial artery was scanned longitudinally 5 to 10 cm above the elbow. Then, a blood pressure cuff was inflated to 50 mmHg above the systolic blood pressure over the proximal portion of the right forearm for 5 min. The diastolic diameter of the brachial artery was determined semi-automatically. The changes in the diastolic diameter were continuously recorded. Then, FMD was determined as the maximum change in diameter after cuff release normalized to the baseline diameter (% of baseline diameter), and measurements of brachial artery diameter were made continuously from 30 s before to at least 2 min after cuff release. The UNEXEF18G consisted of three ultrasound probes with an appropriately flexible stabilizing arm that supports and fixes the ultrasound probes to the human arm. There were two H-shaped ultrasound probes and one longitudinal ultrasound probe, which enabled the technician to easily track target brachial artery images during the FMD study. The UNEXEF18G identifies both the near and far walls of the brachial artery as A-mode ultrasound signals (Figure 2A). The signals of either the intima–media or the main body of brachial artery vessel walls can be re-selected, if necessary, by operators. These signals are automatically chased and recorded continuously to obtain the maximum vasodilation of the brachial artery after the reactive hyperemia Figure 2B. All the measurements are automatically performed with UNEXEF18G. Intra- and inter-observer correlation coefficients were high (>0.9) [8].

### 2.5. Statistical Analysis

The results are expressed as mean ± standard deviation (SD), number (%), or median (inter-quartile interval). Categorical variables were compared using the chi-square test or Fisher’s exact test. Differences in laboratory profiles and endothelial function between the baseline and after 3 months were compared using the paired Student’s *t*-test. Factors independently associated with %FMD or change in %FMD (ΔFMD) were assessed using univariate and multivariate linear regression analyses, and transformed values in logarithm were used as variables for linear regression analyses when the continuous variables did not have a normal distribution. All the analyses used two-sided tests with a significance level of *p* < 0.05. The data were analyzed using SPSS version 25.0 for Windows (SPSS, Inc., Chicago, IL, USA).

## 3. Results

### 3.1. Patient Characteristics

The baseline characteristics of the participants are shown in Table 1. Of the 150 patients, the patients were 70 ± 9 years old and included 116 (77%) men. The mean levels of triglycerides and LDL-C were 127 ± 46 mg/dL and 77 ± 12 mg/dL, respectively. The patients with hypertriglyceridemia (≥150 mg/dL), low EPA/AA levels (<0.3), and both were 76 (51%), 28 (21%), and 68 (45%), respectively.

### 3.2. Serum PUFA Levels and Endothelial Function Inpatients with CAD During Optimal Statin Therapy

The results of the multivariate linear regression analyses of the factors associated with endothelial function assessed by %FMD are shown in Table 2. In the univariate analysis, the age, male gender, diastolic blood pressure, baseline brachial artery diameter, and serum levels of triglyceride and UA were significantly associated with %FMD, but the serum PUFA levels were not significantly associated with %FMD in the patients with CAD during optimal statin therapy and achieved the target LDL-C levels (<100 mg/dL). Moreover, the multivariate linear regression analysis demonstrated that the serum triglyceride, DHA, and EPA plus DHA levels, in addition to age and baseline brachial artery diameter, were independent determinants of %FMD (Model 1: triglyceride, β = −0.486, *p* < 0.0001; DHA, β = 0.214, *p* < 0.05; Model 2: triglyceride, β = −0.401, *p* < 0.0001; EPA plus DHA, β = 0.163, *p* < 0.05; Model 3: triglyceride, β = −0.366, *p* < 0.0001, respectively).

### 3.3. Effects of EPA Therapy on Lipid Profile and Endothelial Function in CAD Patients with High Triglyceride and Low EPA/AA Levels

All the patients were categorized into two groups based on the cutoffs of triglyceride (150 mg/dL) and EPA/AA (0.3): normal triglyceride and high EPA/AA (n = 82) and hypertriglyceridemia and/or low EPA/AA (n = 68). The patients’ baseline characteristics and lipid profiles in each group are shown in Table 1. Regarding endothelial function, %FMD was significantly lower in hypertriglyceridemia and/or low EPA/AA.

**Figure 3:**
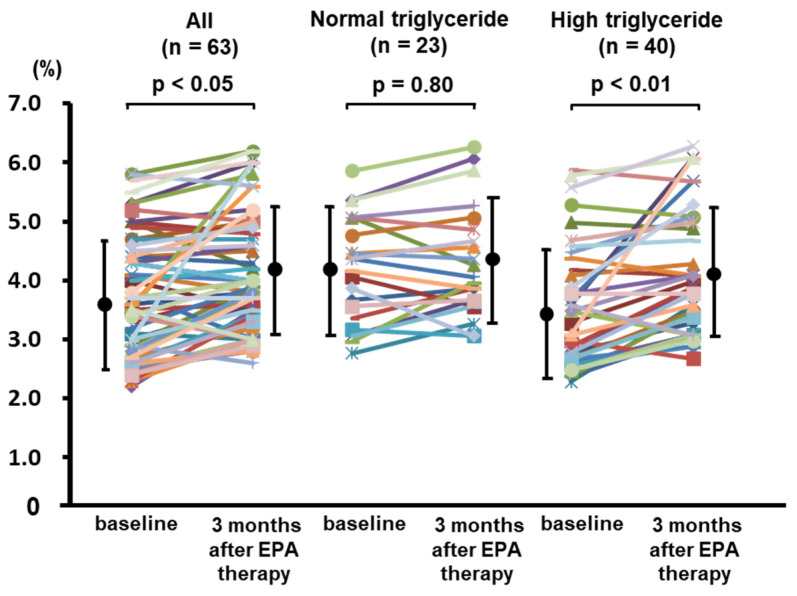
Changes in endothelial function assessed by the flow-mediated dilation (FMD) of the brachial artery between baseline and after 3 months of eicosapentaenoic acid (EPA) therapy. (**Left panel**) all patients (n = 63), (**center panel**) patients with normal triglycerides (n = 23), and (**right panel**) high triglyceride levels (n = 40). Data are expressed as mean ± SD or median (inter-quartile interval).

Finally, the patients with low EPA/AA and normal/high triglyceride (n = 68) were planned to be treated with EPA (1800 mg/day) (Figure 1). Among them, three patients without impaired FMD (<6%) before EPA administration were excluded from the intervention. After starting EPA administration, one patient who stopped taking EPA and one patient who had lost follow-up were withdrawn. Therefore, a total of 63 patients were analyzed. No adverse events occurred during the study. After 3 months, the EPA combination therapy with a statin decreased serum triglyceride (from 167 ± 66 to 141 ± 44 mg/dL, *p* < 0.05) and CRP levels (from 0.19 ± 0.23 to 0.10 ± 0.13 mg/dL, *p* < 0.01) and increased EPA (from 49 ± 24 to 147 ± 36 µg/mL, *p* < 0.0001) and EPA/AA levels (from 0.36 ± 0.21 to 1.20 ± 0.38, *p* < 0.0001) significantly as seen in Table 3. Moreover, the EPA combination therapy significantly improved %FMD (from 3.7 ± 1.0 to 4.1 ± 1.0%, *p* < 0.05) in the patients with low EPA/AA and/or high serum triglyceride levels, especially in the subgroup with high serum triglyceride levels (from 3.4 ± 1.0 to 4.0 ± 1.1%, *p* < 0.01) (Figure 3). In the patients with normal triglyceride levels, EPA therapy did not improve the %FMD (Figure 3). Table 4 shows the results of the univariate and multivariate linear regression analyses of the factors associated with change in %FMD (ΔFMD). ΔFMD was significantly associated with changes in serum triglyceride (β = −0.317, *p* < 0.05) independent of the changes in EPA (β = −0.193, *p* = 0.16), EPA/AA (β = −0.048, *p* = 0.73), and the other profiles in the multivariate analysis.

## 4. Discussion

In this study, we demonstrated that serum DHA and EPA plus DHA levels, in addition to serum triglyceride levels, were significantly associated with %FMD in patients with CAD receiving statin therapy. In addition, the EPA combined with statin therapy improved endothelial function in patients with CAD and low EPA/AA or high triglyceride levels, especially in the high serum triglyceride group.

Yagi et al. reported similar results, noting that a single regression analysis showed no relationships between the %FMD and serum PUFA levels in patients with CAD, but a multiple regression analysis showed a positive relationship between the %FMD and serum DHA levels [18]. That study suggests that a low DHA level is a residual risk factor for endothelial dysfunction in patients with CAD taking statins, and DHA supplementation is a potent therapy for the persistent impairment of FMD despite optimal medical therapy. In addition, Nozue et al. demonstrated that a low serum DHA level, but not EPA, is associated with the progression of coronary atherosclerosis evaluated by intravascular ultrasound with virtual histology in statin-treated patients with DM according to a multivariate stepwise regression analysis [19]. DHA downregulates the expression of pro-inflammatory cytokines on the endothelium [20] and increases the expression and activation of endothelial nitric oxide synthase, resulting in anti-inflammatory actions to endothelial dysfunction [21]. Moreover, DHA supplementation, but not EPA, improves vascular endothelial function in hyperlipidemic overweight men [22]. However, in contrast to the results from this study, EPA has been shown to increase forearm blood flow in response to acetylcholine, an endothelium-dependent vasodilation, in patients with hypertriglyceridaemic [23]. This discrepancy in results regarding the effects of EPA and DHA on endothelial function and cardiovascular outcomes may be due to differences in study design, such as dose, type, duration of omega-3 PUFA, and the characteristics of enrolled patients. These divergent effects on cardiovascular risk markers and the differing clinical effects of EPA and DHA might be explained, at least in part, by differences in the metabolism of EPA to bioactive prostaglandin I_3_ and in the EPA and DHA distributions in phospholipid subclasses [24].

Endothelial dysfunction, as determined by FMD, has been proposed as a predictor of future cardiovascular events [13]. Our group also demonstrated the persistent impairment of FMD (<4.2%) despite optimal medical therapy, which could be associated with the new coronary lesion progression in patients with CAD after PCI in the era of drug-eluting stents [16]. Therefore, an improved strategy for addressing endothelial dysfunction, an integrative “barometer” of vascular conditions, must be considered. Our previous study and the present one showed that hypertriglyceridemia, one of the residual risk factors beyond statin therapy, is an independent factor associated with endothelial dysfunction in patients with CAD during statin therapy. These results might clearly identify the treatment target for residual risk factors [25]. We demonstrated that ezetimibe add-on therapy improved endothelial dysfunction, which was significantly associated with changes in serum triglyceride levels in patients with CAD during statin therapy [25]. Omega-3 PUFAs, such as EPA and DHA, decrease triglyceride levels by inhibiting very-low-density lipoprotein synthesis in the liver and increasing the clearance of plasma triglycerides. The potential beneficial effects of EPA and DHA on cardiovascular risk reduction are well recognized, and both have potent triglyceride-lowering effects [24]. However, two meta-analyses found that EPA lowered triglycerides without adversely affecting LDL-C, whereas DHA decreased triglycerides but raised LDL-C [26,27]. This study showed that 3 months of EPA combination therapy with a statin did not affect the serum LDL-C levels. Moreover, Itakura et al. analyzed the correlation between the absolute change in plasma fatty acid concentrations and serum lipids in Japan EPA Lipid Intervention Study (JELIS) participants, reporting changes in plasma DHA, but not EPA, concentrations were positively correlated with changes in LDL-C [28]. According to the available evidence, EPA therapy may provide clinical benefits in lowering triglycerides without interfering with reaching or maintaining the target LDL-C levels.

In the current JAS guidelines 2022, EPA is recommended to indicate the treatment of dyslipidemia accompanied by increased triglyceride, but the EPA recommendation is undescribed regarding the serum EPA or EPA/AA levels [29]. In primary prevention, Ninomiya et al. demonstrated that a lower serum EPA/AA ratio (<0.29) was significantly associated with an increased risk of CAD in the HISAYAMA study [30]. In secondary prevention for CAD or peripheral artery disease, a lower EPA/AA ratio (<0.30–0.40) had a significantly higher incidence of major adverse cardiac events [31,32]. In the present study, EPA combined with statin therapy improved endothelial function in CAD patients with low EPA/AA and/or high triglyceride levels, especially in the high serum triglyceride group. Moreover, the improvement in endothelial function was significantly associated with serum triglyceride reduction. A previous report from a sub-analysis of JELIS has also reconfirmed non-HDL-C as a residual risk marker of CAD after LDL-C-lowering therapy and EPA treatment suppressed the risk of CAD by 53% in these higher-risk patients with high triglyceride with low HDL-C [33,34]. Our data and those previous data strongly suggest that a lower EPA/AA ratio (<0.30–0.40) complicating with high triglyceride/low HDL-C levels should be recommended when considering candidates for EPA treatment in secondary prevention. A further, large-scale, randomized trial is needed to clarify this point.

This study had some limitations. First, this study was conducted between 2016 and 2017 and included patients with LDL-C < 100 mg/dL, the target level according to the JAS guideline 2013 [17]. Therefore, the results of this study may not be directly applicable to the current clinical practice. Second, this study is a preliminary, single-arm study and not a randomized controlled trial. Large randomized studies are needed to confirm our findings. Third, the number of patients enrolled was relatively small, and selection bias due to inclusion by the attending physicians should be considered. Fourth, factors such as exercise, diet therapy, and several pharmacological agents often affect endothelial function, and their influence on FMD values cannot be ruled out. However, to minimize the effect of related factors, the enrolled patients did not receive other drugs during the study.

## 5. Conclusions

Serum DHA and EPA plus DHA levels, in addition to serum triglyceride levels, were associated with endothelial dysfunction in patients with CAD under statin therapy (LDL-C levels < 100 mg/dL). Measurements of serum PUFA concentrations may be useful for identifying high-risk patients, and EPA combination therapy with optimal statin treatment may improve endothelial function in selected high-risk populations with CAD and low EPA/AA or high triglyceride levels. However, prospective, large randomized trials are needed to investigate whether treatment with EP improves the prognosis of statin-treated CAD patients with low EPA/AA and high triglyceride levels.

## Figures and Tables

**Table 1:** Baseline clinical characteristics according to the EPAA/AA.

Variable	All	High EPA/AA (≥ 0.3)	Low EPA/AA (<0.3)	*p*-Value
		(n = 82)	(n = 68)	
Age (years)	70 ± 9	70 ± 9	68 ± 8	0.09
Men	116 (77%)	60 (73%)	56 (82%)	0.18
BMI (kg/m^2^)	24.1 ± 3.5	23.6 ± 3.7	24.7 ± 3.2	0.07
Hypertension	112 (75%)	60 (73%)	52 (76%)	0.64
Diabetes mellitus	74 (49%)	38 (46%)	36 (53%)	0.42
Current smoker	110 (73%)	55 (67%)	55 (81%)	0.06
Angina pectoris/OMI	136 (90%)/34 (23%)	68 (45%)/14 (9%)	48 (32%)/20 (13%)	0.07
Multivessel disease	79 (52%)	38 (46%)	41 (60%)	0.09
CABG	8 (5%)	3 (4%)	5 (7%)	0.32
Cerebral vascular disease	21 (14)	12 (15%)	9 (13%)	0.81
Peripheral artery disease	14 (9%)	6 (7%)	8 (12%)	0.35
Antiplatelet	150 (100%)	82 (100%)	68 (100%)	> 0.99
ACEi or ARB	113 (75%)	61 (74%)	52 (76%)	0.77
β-blocker	82 (55%)	45 (55%)	37 (54%)	0.95
Statin	119 (79%)	82 (100%)	68 (100%)	> 0.99
CCB	65 (43%)	32 (39%)	33 (49%)	0.24
Nitrates	21 (14%)	12 (15%)	9 (13%)	0.88
Ezetimibe	14 (9%)	7 (9%)	7 (10%)	0.71
Sulfonylurea	21 (14%)	11 (13%)	10 (15%)	0.82
Insulin	16 (11%)	9 (11%)	7 (10%)	0.89
Triglyceride (mg/dL)	127 ± 46	96 ± 30	164 ± 65	<0.0001
LDL cholesterol (mg/dL)	77 ± 12	76 ± 13	79 ± 10	0.11
HDL cholesterol (mg/dL)	43 ± 11	46 ± 12	40 ± 9	<0.001
Uric acid (mg/dL)	6.1 ± 1.6	6.1 ± 1.6	6.2 ± 1.7	0.56
FBG (mg/dL)	126 ± 44	121 ± 43	133 ± 46	<0.005
HemoglobinA1c (%)	5.9 ± 0.9	5.8 ± 0.8	6.1 ± 1.2	0.64
hsCRP (mg/dL)	0.30 ± 0.53	0.41 ± 0.79	0.18 ± 0.23	0.77
eGFR (ml/min/1.73 m^2^)	62.0 ± 21.7	62.9 ± 21.8	61.0 ± 21.7	0.61
EPA (μg/mL)	44.9 (29.4–68.2)	72.5 (53.8–81.0)	30.4 (25.8–43.7)	<0.001
AA (μg/mL)	134.6 (110.9–182.0)	124.8 (101.2–145.2)	150.0 (119.7–193.9)	<0.001
DHA (μg/mL)	112.8 (80.2–144.2)	140.5 (97.4–159.8)	96.8 (63.5–132.6)	<0.001
DHLA (μg/mL)	29.8 (22.8–37.6)	30.1 (25.6–35.4)	29.2 (21.2–38.0)	0.72
Baseline brachial artery diameter (mm)	4.04 ± 0.48	3.99 ± 0.53	4.12 ± 0.43	0.19
FMD (%)	4.1 ± 1.3	4.3 ± 1.3	3.9 ± 1.3	<0.05

Data are expressed as mean ± SD, number (percentage), or median (inter-quartile interval). OMI, old myocardial infarction; CABG, coronary artery bypass grafting; ACEi, angiotensin-converting enzyme inhibitor; ARB, angiotensin receptor blocker; CCB, calcium channel blocker; LDL, low-density lipoprotein; HDL, high-density lipoprotein; FBG, fasting blood glucose; hsCRP, high-sensitivity C reactive protein; EPA, eicosapentaenoic acid: DHA, docosahexaenoic acid; AA, arachidonic acid; DHLA, dihomogammalinolenic acid. FMD, flow-mediated dilation.

**Table 2:** Univariate and multivariate linear regression analyses for determinants of % flow-mediated dilation (n = 150).

Variable	Univariate Analysis		Multivariate Analysis	
	r	*p*-Value	β	*p*-Value
**Model 1**				
Age, years	−0.277	<0.001	−0.277	<0.001
Men	−0.224	<0.01	−0.031	0.69
Body mass index	−0.132	0.11		
Systolic blood pressure	0.036	0.66		
Diastolic blood pressure	0.167	<0.05	0.181	<0.05
Baseline brachial artery diameter *	−0.293	<0.001	−0.148	<0.05
Triglyceride *	−0.277	<0.001	−0.486	<0.0001
Low-density lipoprotein cholesterol	0.088	0.28		
High-density lipoprotein cholesterol *	0.151	0.06	−0.003	0.97
Uric acid *	−0.248	<0.01	−0.121	0.10
Fasting blood glucose *	−0.064	0.43		
Hemoglobin A1c *	−0.074	0.38		
C-reactive protein *	0.023	0.78		
EPA *	0.111	0.18	−0.024	0.79
DHA *	0.142	0.08	0.214	<0.05
AA *	0.100	0.22	−0.011	0.90
DHLA*	0.031	0.71	0.112	0.25
**Model 2**				
Age, years	−0.277	<0.001	−0.260	<0.01
Men	−0.224	<0.01	−0.735	0.46
Diastolic blood pressure	0.167	<0.05	0.181	<0.05
Baseline brachial artery diameter *	−0.293	<0.001	−0.160	<0.05
Triglyceride *	−0.277	<0.001	−0.401	<0.0001
High-density lipoprotein cholesterol *	0.151	0.06	0.010	0.90
Uric acid *	−0.248	<0.01	−0.130	0.08
EPA+DHA *	0.115	0.16	0.163	<0.05
AA+DHLA *	0.129	0.12	0.054	0.51
**Model 3**				
Age, years	−0.277	<0.001	−0.276	<0.001
Men	−0.224	<0.01	−0.082	0.28
Diastolic blood pressure	0.167	<0.05	0.190	<0.05
Baseline brachial artery diameter *	−0.293	<0.001	−0.166	<0.05
Triglyceride *	−0.277	<0.001	−0.366	<0.0001
High-density lipoprotein cholesterol *	0.151	0.06	0.058	0.48
Uric acid *	−0.248	<0.01	−0.139	0.06
EPA/AA	−0.005	0.95	−0.069	0.49
DHA/AA	−0.038	0.65	0.154	0.13

Multivariate analysis included significant factors (*p* < 0.1) of the univariate analysis and serum polyunsaturated fatty acid levels. * = Logarithmically transformed. EPA, eicosapentaenoic acid; DHA, docosahexaenoic acid; AA, rachidonic acid; DHLA, dihomogammalinolenic acid.

**Table 3:** Laboratory data at baseline and 3 months after EPA administration.

Variables	Baseline	3 Months	*p*-Value
Triglyceride (mg/dL)	167 ± 66	141 ± 44	<0.05
LDL cholesterol (mg/dL)	79 ± 11	76 ± 8	0.06
HDL cholesterol (mg/dL)	40 ± 9	41 ± 7	0.20
Uric acid (mg/dL)	6.3 ± 1.5	6.3 ± 1.7	0.93
Fasting blood glucose (mg/dL)	134 ± 47	126 ± 29	0.54
HemoblobinA1c (%)	6.1 ± 1.2	6.1 ± 1.0	0.76
hsCRP (mg/dL)	0.19 ± 0.23	0.10 ± 0.13	<0.01
EPA (μg/mL)	49 ± 24	147 ± 36	<0.0001
AA (μg/mL)	146 ± 47	130 ± 38	<0.05
DHA (μg/mL)	115 ± 45	109 ± 42	0.49
EPA/AA	0.36 ± 0.21	1.20 ± 0.38	<0.0001
DHA/AA	0.84 ± 0.36	0.88 ± 0.37	0.48

Data are expressed as mean ± SD. Abbreviations are as in Table 1.

**Table 4:** Univariate and multivariate linear regression analyses for predicting change in %FMD (ΔFMD) after 3 months of EPA therapy.

Variables	Univariate Analysis		Multivariate Analysis	
	r	*p*-Value	β	*p*-Value
ΔTG	−0.321	<0.05	−0.317	<0.05
ΔLDL cholesterol	−0.015	0.91		
ΔHDL cholesterol	0.022	0.86		
ΔUric acid	0.021	0.87		
ΔFasting blood glucose	−0.098	0.45		
ΔHemoglobinbA1c	−0.162	0.21		
ΔCRP	−0.032	0.81		
ΔEPA	−0.218	0.09	−0.193	0.16
ΔAA	0.050	0.69		
ΔDHA	0.019	0.88		
ΔEPA/AA	−0.170	0.18	−0.048	0.73
ΔDHA/AA	0.023	0.86		

The multivariate analysis included the significant factors (*p* < 0.1) of the univariate analysis and ΔEPA/AA. Abbreviations are as in Table 1.

## Data Availability

The data presented in this study are available upon request from the corresponding author. The data are not publicly available due to privacy restrictions.

## References

[1] Arnett D.K., Blumenthal R.S., Albert M.A., Buroker A.B., Goldberger Z.D., Hahn E.J., Himmelfarb C.D., Khera A., Lloyd-Jones D., McEvoy J.W. (2019). 2019 ACC/AHA Guideline on the primary prevention of cardiovascular disease: A report of the american college of cardiology/American heart association task force on clinical practice guidelines. Circulation.

[2] (1994). Scandinavian Simvastatin Survival Study Group Randomized trial of cholesterol lowering in 4444 patients with coronary heart disease: The Scandinavian Simvastatin Survival Study (4S). Lancet.

[3] (1998). Long-Term Intervention with Pravastatin in Ischaemic Disease (LIPID) Study Group Prevention of Cardiovascular Events and Death with Pravastatin in Patients with Coronary Heart Disease and a Broad Range of Initial Cholesterol Levels. N. Engl. J. Med..

[4] LaRosa J.C., He J., Vupputuri S. (1999). Effect of Statins on Risk of Coronary Disease. JAMA.

[5] German C.A., Liao J.K. (2023). Understanding the molecular mechanisms of statin pleiotropic effects. Arch. Toxicol..

[6] Ganda O.P., Bhatt D.L., Mason R.P., Miller M., Boden W.E. (2018). Unmet Need for Adjunctive Dyslipidemia Therapy in Hypertriglyceridemia Management. J. Am. Coll. Cardiol..

[7] Liao J., Xiong Q., Yin Y., Ling Z., Chen S. (2022). The Effects of Fish Oil on Cardiovascular Diseases: Systematical Evaluation and Recent Advance. Front. Cardiovasc. Med..

[8] Miyoshi T., Noda Y., Ohno Y., Sugiyama H., Oe H., Nakamura K., Kohno K., Ito H. (2014). Omega-3 fatty acids improve postprandial lipemia and associated endothelial dysfunction in healthy individuals—a randomized cross-over trial. Biomed. Pharmacother..

[9] Bhatt D.L., Steg P.G., Miller M., Brinton E.A., Jacobson T.A., Ketchum S.B., Doyle R.T., Juliano R.A., Jiao L., Granowitz C. (2019). Cardiovascular Risk Reduction with Icosapent Ethyl for Hypertriglyceridemia. N. Engl. J. Med..

[10] Nicholls S.J., Lincoff A.M., Garcia M., Bash D., Ballantyne C.M., Barter P.J., Davidson M.H., Kastelein J.J.P., Koenig W., McGuire D.K. (2020). Effect of High-Dose Omega-3 Fatty Acids vs Corn Oil on Major Adverse Cardiovascular Events in Patients at High Cardiovascular Risk. JAMA.

[11] Miyauchi K., Iwata H., Nishizaki Y., Inoue T., Hirayama A., Kimura K., Ozaki Y., Murohara T., Ueshima K., Kuwabara Y. (2024). Randomized Trial for Evaluation in Secondary Prevention Efficacy of Combination Therapy–Statin and Eicosapentaenoic Acid (RESPECT-EPA). Circulation.

[12] Alexander Y., Osto E., Schmidt-Trucksäss A., Shechter M., Trifunovic D., Duncker D.J., Aboyans V., Bäck M., Badimon L., Cosentino F. (2021). Endothelial function in cardiovascular medicine: A consensus paper of the European Society of Cardiology Working Groups on Atherosclerosis and Vascular Biology, Aorta and Peripheral Vascular Diseases, Coronary Pathophysiology and Microcirculation, and Thrombosis. Cardiovasc. Res..

[13] Matsuzawa Y., Kwon T., Lennon R.J., Lerman L.O., Lerman A. (2015). Prognostic Value of Flow-Mediated Vasodilation in Brachial Artery and Fingertip Artery for Cardiovascular Events: A Systematic Review and Meta-Analysis. J. Am. Hear. Assoc..

[14] Corretti M.C., Anderson T.J., Benjamin E.J., Celermajer D., Charbonneau F., Creager M.A., Deanfield J., Drexler H., Gerhard-Herman M., Herrington D. (2002). Guidelines for the ultrasound assessment of endothelial-dependent flow-mediated vasodilation of the brachial artery: A report of the International Brachial Artery Reactivity Task Force. J. Am. Coll. Cardiol..

[15] Kitta Y., Obata J.-E., Nakamura T., Hirano M., Kodama Y., Fujioka D., Saito Y., Kawabata K.-I., Sano K., Kobayashi T. (2009). Persistent Impairment of Endothelial Vasomotor Function Has a Negative Impact on Outcome in Patients With Coronary Artery Disease. Circ..

[16] Kubo M., Miyoshi T., Oe H., Ohno Y., Nakamura K., Ito H. (2015). Prognostic significance of endothelial dysfunction in patients undergoing percutaneous coronary intervention in the era of drug-eluting stents. BMC Cardiovasc. Disord..

[17] Teramoto T., Sasaki J., Ishibashi S., Birou S., Daida H., Dohi S., Egusa G., Hiro T., Hirobe K., Iida M. (2013). Executive Summary of the Japan Atherosclerosis Society (JAS) Guidelines for the Diagnosis and Prevention of Atherosclerotic Cardiovascular Diseases in Japan ^|^mdash;2012 Version. J. Atheroscler. Thromb..

[18] Yagi S., Aihara K.-I., Fukuda D., Takashima A., Hara T., Hotchi J., Ise T., Yamaguchi K., Tobiume T., Iwase T. (2015). Effects of Docosahexaenoic Acid on the Endothelial Function in Patients with Coronary Artery Disease. J. Atheroscler. Thromb..

[19] Nozue T., Yamamoto S., Tohyama S., Fukui K., Umezawa S., Onishi Y., Kunishima T., Sato A., Nozato T., Miyake S. (2014). Low serum docosahexaenoic acid is associated with progression of coronary atherosclerosis in statin-treated patients with diabetes mellitus: Results of the treatment with statin on atheroma regression evaluated by intravascular ultrasound with virtual histology (TRUTH) study. Cardiovasc. Diabetol..

[20] Richard D., Wolf C., Barbe U., Kefi K., Bausero P., Visioli F. (2009). Docosahexaenoic acid down-regulates endothelial Nox 4 through a sPLA2 signalling pathway. Biochem. Biophys. Res. Commun..

[21] Chen J., Shearer G.C., Chen Q., Healy C.L., Beyer A.J., Nareddy V.B., Gerdes A.M., Harris W.S., O’Connell T.D., Wang D. (2011). Omega-3 Fatty Acids Prevent Pressure Overload–Induced Cardiac Fibrosis Through Activation of Cyclic GMP/Protein Kinase G Signaling in Cardiac Fibroblasts. Circulation.

[22] Mori T.A., Watts G.F., Burke V., Hilme E., Puddey I.B., Beilin L.J. (2000). Differential Effects of Eicosapentaenoic Acid and Docosahexaenoic Acid on Vascular Reactivity of the Forearm Microcirculation in Hyperlipidemic, Overweight Men. Circulation.

[23] Okumura T., Fujioka Y., Morimoto S., Tsuboi S., Masai M., Tsujino T., Ohyanagi M., Iwasaki T. (2002). Eicosapentaenoic Acid Improves Endothelial Function in Hypertriglyceridemic Subjects Despite Increased Lipid Oxidizability. Am. J. Med Sci..

[24] Ohnishi H., Saito Y. (2013). Eicosapentaenoic Acid (EPA) Reduces Cardiovascular Events: Relationship with the EPA/Arachidonic Acid Ratio. J. Atheroscler. Thromb..

[25] Yunoki K., Nakamura K., Miyoshi T., Enko K., Kubo M., Murakami M., Hata Y., Kohno K., Morita H., Kusano K.F. (2011). Impact of Hypertriglyceridemia on Endothelial Dysfunction During Statin ± Ezetimibe Therapy in Patients With Coronary Heart Disease. Am. J. Cardiol..

[26] Jacobson T.A., Glickstein S.B., Rowe J.D., Soni P.N. (2012). Effects of eicosapentaenoic acid and docosahexaenoic acid on low-density lipoprotein cholesterol and other lipids: A review. J. Clin. Lipidol..

[27] Wei M.Y., Jacobson T.A. (2011). Effects of Eicosapentaenoic Acid Versus Docosahexaenoic Acid on Serum Lipids: A Systematic Review and Meta-Analysis. Curr. Atheroscler. Rep..

[28] Itakura H., Yokoyama M., Matsuzaki M., Saito Y., Origasa H., Ishikawa Y., Oikawa S., Sasaki J., Hishida H., Kita T. (2012). The Change in Low-Density Lipoprotein Cholesterol Concentration is Positively Related to Plasma Docosahexaenoic Acid but not Eicosapentaenoic Acid. J. Atheroscler. Thromb..

[29] Okamura T., Tsukamoto K., Arai H., Fujioka Y., Ishigaki Y., Koba S., Ohmura H., Shoji T., Yokote K., Yoshida H. (2024). Japan Atherosclerosis Society (JAS) Guidelines for Prevention of Atherosclerotic Cardiovascular Diseases 2022. J. Atheroscler. Thromb..

[30] Ninomiya T., Nagata M., Hata J., Hirakawa Y., Ozawa M., Yoshida D., Ohara T., Kishimoto H., Mukai N., Fukuhara M. (2013). Association between ratio of serum eicosapentaenoic acid to arachidonic acid and risk of cardiovascular disease: The Hisayama Study. Atherosclerosis.

[31] Domei T., Yokoi H., Kuramitsu S., Soga Y., Arita T., Ando K., Shirai S., Kondo K., Sakai K., Goya M. (2012). Ratio of Serum n-3 to n-6 Polyunsaturated Fatty Acids and the Incidence of Major Adverse Cardiac Events in Patients Undergoing Percutaneous Coronary Intervention. Circ. J..

[32] Hishikari K., Kimura S., Yamakami Y., Kojima K., Sagawa Y., Otani H., Sugiyama T., Kuwahara T., Hikita H., Takahashi A. (2015). The prognostic value of the serum eicosapentaenoic acid to arachidonic acid ratio in relation to clinical outcomes after endovascular therapy in patients with peripheral artery disease caused by femoropopliteal artery lesions. Atherosclerosis.

[33] Sasaki J., Yokoyama M., Matsuzaki M., Saito Y., Origasa H., Ishikawa Y., Oikawa S., Itakura H., Hishida H., Kita T. (2012). Relationship between Coronary Artery Disease and Non-HDL-C, and Effect of Highly Purified EPA on the Risk of Coronary Artery Disease in Hypercholesterolemic Patients Treated with Statins: Sub-Analysis of the Japan EPA Lipid Intervention Study (JELIS). J. Atheroscler. Thromb..

[34] Saito Y., Yokoyama M., Origasa H., Matsuzaki M., Matsuzawa Y., Ishikawa Y., Oikawa S., Sasaki J., Hishida H., Itakura H. (2008). Effects of EPA on coronary artery disease in hypercholesterolemic patients with multiple risk factors: Sub-analysis of primary prevention cases from the Japan EPA Lipid Intervention Study (JELIS). Atherosclerosis.

